# Effects of Harvest Time on the Aroma of White Wines Made from Cold-Hardy Brianna and Frontenac Gris Grapes Using Headspace Solid-Phase Microextraction and Gas Chromatography-Mass Spectrometry-Olfactometry

**DOI:** 10.3390/foods8010029

**Published:** 2019-01-16

**Authors:** Somchai Rice, Madina Tursumbayeva, Matthew Clark, David Greenlee, Murlidhar Dharmadhikari, Anne Fennell, Jacek A. Koziel

**Affiliations:** 1 Midwest Grape and Wine Industry Institute, Iowa State University, Ames, IA 50011, USA; somchai@iastate.edu (S.R.); murli@iastate.edu (M.D.); 2 Interdepartmental Toxicology Graduate Program, Iowa State University, Ames, IA 50011, USA; 3 Department of Agricultural and Biosystems Engineering, Iowa State University, Ames, IA 50011, USA; madina@iastate.edu; 4 Department for Horticultural Science, University of Minnesota, St. Paul, MN 55108, USA; clark776@umn.edu; 5 Tucker’s Walk Vineyard and Winery, Garretson, SD 57030, USA; dave@tuckerswalk.com; 6 Department of Agronomy, Horticulture and Plant Science, BioSNTR, South Dakota State University, Brookings, SD 57006, USA; anne.fennell@sdstate.edu

**Keywords:** Frontenac gris, Brianna, wine aroma, SPME-GC-MS, olfactometry, cold-hardy grapes

## Abstract

The Midwest wine industry has shown a marked increase in growers, hectares planted, wineries, and wine production. This growth coincides with the release of cold-hardy cultivars such as Brianna and Frontenac gris, in 2001 and 2003, respectively. These white grape varieties account for one-third of the total area grown in the state of Iowa. It is generally accepted that the wine aroma profile plays a crucial role in developing a local, sustainable brand. However, the identity of Brianna/Frontenac Gris-based wine aromas and their link to the grape berry chemistry at harvest is unknown. This study aims to preliminarily characterize key odor-active compounds that can influence the aroma profile in wines made from Brianna and Frontenac gris grapes harvested at different stages of ripening. Brianna and Frontenac gris grapes were harvested approximately 7 days apart, starting at 15.4 °Brix (3.09 pH) and 19.5 °Brix (3.00 pH), respectively. Small batch fermentations were made for each time point with all juices adjusted to the same °Brix prior to fermentation. Odor-active compounds were extracted from wine headspace using solid-phase microextraction (SPME) and analyzed by gas chromatography-mass spectrometry (GC-MS) and simultaneous olfactometry (O). Over 30 odor-active compounds were detected. Aromas in Brianna wines developed from “cotton candy” and “floral”, to “banana” and “butterscotch”, then finally to “honey”, “caramel” and an unknown neutral aroma. Frontenac gris wines changed from an unknown neutral aroma to “fruity” and “rose”. Results from the lay audiences’ flavor and aroma descriptors also indicate a shift with harvest date and associated °Brix. To date, this is the first report of wine aromas from Brianna and Frontenac gris by GC-MS-O. Findings from this research support the hypothesis that aroma profiles of Brianna and Frontenac gris wines can be influenced by harvesting the grapes at different stages of ripening.

## 1. Introduction

The business of grapes and wine generated over $7.5 billion U.S. dollars (USD) in the upper Midwestern states of Iowa, Illinois, Michigan, Minnesota, North Dakota, South Dakota, and Wisconsin in 2017 [1]. This included the direct economic impact from vineyard and winery activities as well as tourism, resulting in over 110,000 jobs and over $3 billion in wages (Table 1).

**Figure 1:**
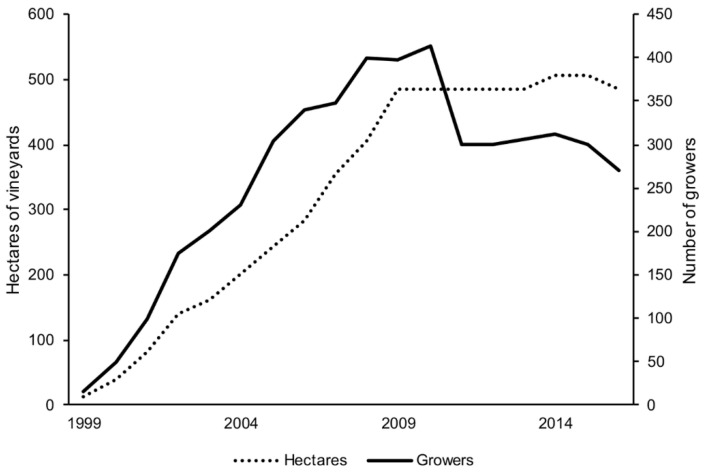
The increase in hectares of wine grapes and the number of growers in Iowa [2].

**Figure 2:**
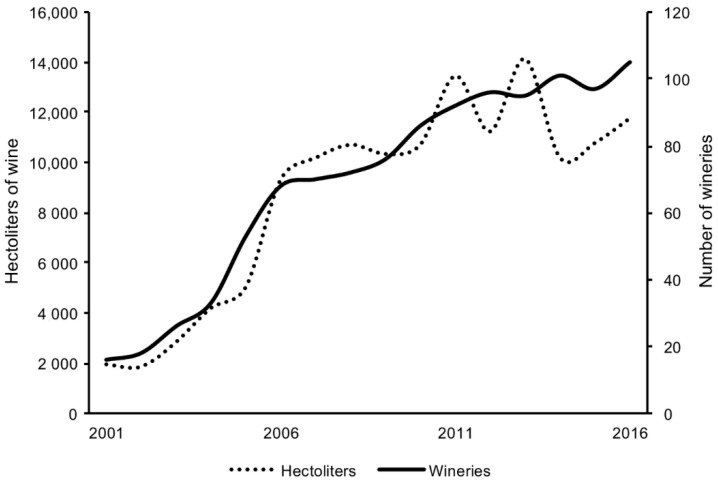
The increase in wine production (hectoliters) and a number of wineries in Iowa [2].

In Iowa, the number of grape growers, vineyards of grapes, wineries, and wine production has increased in the last two decades (Figure 1 and Figure 2) [2]. In a report by Tuck and Gartner in 2014, 100 hectares of grapes planted in Iowa were of the cold-hardy white varieties [3]. These numbers were extrapolated from self-reported surveys to determine the baseline of activity involving cold-hardy grape varieties. Of this estimated 100 hectares, 27% of the plantings were Frontenac gris and Brianna varieties.

There is continuous interest in understanding the chemical origin of grape aromas. Our working hypothesis is that this information could help growers and winemakers to determine a more targeted harvest date, based on the desired aromas. It also would allow an assessment of how various winemaking practices influence aroma, an important factor of wine quality. This information could streamline the production of new grape varieties by permitting the selection of varieties showing certain aromatic attributes. Despite these advantages, determining the chemical origin of varietal aromatic character is complicated. First, odor-active compounds in grapes often occur in nonvolatile forms. These compounds are released only upon crushing [4], through yeast metabolism [5], or during aging [6]. A varietal character can originate from a combination of compounds and not from varietal specific compounds. Extraction procedures may influence the stability of odor-active compounds. Identification and quantification of odor-active compounds are needed to understand the aroma potential of new cold-hardy grape varieties.

The added benefit of simultaneous olfactometry (O) and chemical analysis (e.g., by gas chromatography-mass spectrometry (GC-MS)) allows for characterization of trace amounts of compounds with detection limits below that of the mass detector (i.e., 2-isobutyl-3-methoxypyrazine, a “green bell pepper” aroma with detection threshold less than 0 ppb) [7,8]. Brianna is known, at least anecdotally within the industry, to develop an unwanted “foxy” aroma if harvested after 14–16 °Brix. However, there is a lack of scientific data to support this observation.

Grape maturity levels expressed by sugar content (measured as °Brix) and titratable acidity (TA) in grape berries has a great impact on wine quality and aroma as well. During the period of grape ripening, sugar content increases in berries while the TA level decreases. The relationship between those two factors affects the release of wine odor-active compounds. As sugar increases and acidity decreases, the aroma of wine changes from “herbal” to “fruity” [9]. However, higher sugar content in berries resulting in higher ethanol production can decrease the volatility of odor-active compounds in wine, and fruity aromas change to alcohol-associated aromas [10]. Grapes are typically harvested when pH levels are between 3.2 to 3.4 for Brianna [11] and around 3.0 for Frontenac gris [12]. Brianna fruit has “grapefruit, tropical” and slight “floral” characteristics [11]. Frontenac gris fruit has aromas of “peach, apricot” and “tropical fruits” [13]. These cold-hardy cultivars were introduced to the public in 2001 (Brianna) [11] and 2003 (Frontenac gris) [12]. The cultivars are advantageous in cold climates, where *V. vinifera* will not survive the extreme low temperatures. Brianna was shown to be a top yielding cultivar among select cold-hardy cultivars with the lowest average titratable acidity [14].

There is a need to characterize aromas from these new cold-hardy cultivars in order to understand and improve the potential of the final product. The objective of this study was to preliminarily associate odor-active compounds in Brianna and Frontenac gris white wines with different stages of grape berry ripening (i.e., with increasing sugar content and pH). This was completed by analyzing odor-active compounds in the headspace of wine using solid-phase microextraction (SPME) and simultaneous chemical and sensory analysis using gas chromatography-mass spectrometry-olfactometry (GC-MS-O) [15,16].

In our previous research, we developed an automated headspace SPME-GC-MS-O method for aroma profiles of seven cold-hardy wines [15]. The effects of the SPME fiber type (7 coatings), the headspace SPME extraction time (10 distinct times from 10 s to 1 h), the extraction temperature (6 set points from 35 to 80 °C), the incubation time (5 set points for headspace equilibration from 0 to 20 min), the sample volume (4 set points from 1 to 4 mL in a 10 mL vial), the desorption time (6 set points from 30 to 300 s), and the salt addition (5 set points) were tested. We used the optimized SPME conditions from previous research [15] in this current work. A multivariate analysis was used to illustrate the effects of harvest time on wine odor-active compounds. There is a need to characterize aromas from these new cold-hardy cultivars in order to understand and improve the potential of the final product. This is the first report of odor-active compounds in wines made from Frontenac gris and Brianna grapes at different levels of maturity. Information from this study can guide growers and winemakers in optimizing winemaking techniques and harvest decisions. This (GC-O) technique has been used in wine aroma analysis in Chardonnay [17], Muscat [18], Cabernet Gernischt, Cabernet Sauvignon, Cabernet Franc, Merlot [19], and native American grapes (Vitis) [20].

## 2. Materials and Methods

### 2.1. Grape Samples Collection and Winemaking

The working hypothesis is that wine aromas are affected by Brianna and Frontenac gris berry maturation (i.e., change in pH and sugar content as °Brix) at the time of harvest. Brianna and Frontenac gris grapes were grown in a Tucker’s Walk vineyard in Garretson, South Dakota. Brianna and Frontenac gris grapes’ characteristics at harvest are given in Table 2. Tucker’s Walk produced the wines using the protocols developed for the Northern Grapes Project [21] during the 2015 growing season and are described as follows. Briefly, grapes were harvested approximately one week apart. Four small batches of Brianna and three small batches of Frontenac gris wines were made on-site, (*n* = 2), using the same winemaking process. Grapes (110 to 120 kg) were processed in a crusher/destemmer and pressed, and juice sugar content was adjusted to 24 °Brix for Frontenac gris and 20 °Brix for Brianna. Frontenac gris, a bud sport from Frontenac and a high acid grape, is typically harvested for commercial wine at 22–24 °Brix. Brianna, a low acid grape, is typically harvested between 16 and 20 Brix. Brianna and Frontenac gris juices were brought to 20 and 24 °Brix, respectively, at each harvest time point and fermented to dryness. This provided the same alcohol content in the respective cultivars across harvest dates. Inoculated juice was allowed to start fermenting at ambient temperature for 24 h, then immediately moved into 13 °C and fermented to dryness. The wines (14 total) were analyzed by chemical and sensory analysis in triplicate.

### 2.2. Informal Sensory Analysis of Brianna Wine by Wine Industry Professionals

Wines from each fermentation were analyzed in blind tastings by lay audiences at two viticulture and enology conferences (Minnesota Cold Climate Conference and Nebraska Vindemia). These panelists included grape growers, winemakers, vineyard/winery owners, and research scientists. Data was gathered from 32 and 23 individuals, respectively, and pooled for analysis. The panelists were asked to provide flavor descriptors and any wine quality notes. The descriptive terms were generated by the audience members and extracted from the data sheets. The terms were reduced from 78 to 61 terms by combining similar terms. For example, “citrus” includes lemon, lemongrass, grapefruit, and lime. The top 24 terms were selected as those having been mentioned by at least 4 panelists. A spider plot was created using the term’s incidence as the response variable.

### 2.3. Preparation of Wine Samples

A 10 mL glass amber vial with a magnetic screw top and polytetrafluoroethylene (PTFE)-lined septum was used. Undiluted wine samples and serial dilutions of wine samples in model wine (4 mL total volume) were prepared using dilution factors of 2, 4, 8, 16, and 32 [22]. The model wine was 5 mg/mL of potassium bitartrate in 12% ethanol in water. Two g of sodium chloride was added to each 10 mL vial to enhance headspace SPME extraction.

### 2.4. Automated SPME Extraction

A 50/30 µm divinylbenzene (DVB)/Carboxen/polydimethylsiloxane (PDMS) SPME fiber (Sigma-Aldrich, St. Louis, MO, USA) was used to extract and pre-concentrate odor-active compounds from the headspace of wine samples. A Leap Technologies CombiPal (Trajan Scientific, Pflugerville, TX, USA) was used for automated headspace sampling with the following parameters: 500 rpm agitation speed during incubation and extraction, 10 min incubation/extraction time at 50 °C, and 260 °C desorption for 2 min directly into the GC inlet. To prevent carryover between samples, the SPME fiber was also cleaned in a needle heater (260 °C for 2 min) under a flow of clean helium prior to each analysis.

### 2.5. Chemical and Sensory Analysis

An Agilent (7890B and 5977A) GC-MS was used for analysis, fitted with two columns in series. The first column was non-polar (BPX-2, 83 m × 530 µm × 0.5 µm, SGE-Trajan Scientific, Pflugerville, TX, USA) and pressure balanced at the midpoint with a second polar column (DB-WAXETR, 30 m × 530 µm × 0.25 µm, Agilent Technologies, Santa Clara, CA, USA). Effluent from the second column was split 1:3 by restrictor columns to the single quadrupole mass spectrometer and olfactometry sniff port, respectively (1 part to MS and 2 parts to O-port). The GC temperature profile was initially 40 °C, held for 3 min, 7 °C/min ramp to 220 °C, held for 11.29 min. Data acquisition was collected in full scan mode, the mass range was m/z 33 to 450, and the electron ionization energy was 70 eV. The instrument was tuned daily prior to analysis. MassHunter (v. B.07.00.1413, Agilent, Santa Clara, CA, USA) was used for mass spectral data acquisition and analysis. AromaTrax (v. 10.1, MOCON, Round Rock, TX, USA) was used for sensory data acquisition (i.e., the aromagram). Multitrax Multidimensional Control Software (v. 10.1, MOCON, Round Rock, TX, USA) was used for pressure balance programming. A single trained human panelist was used to assign aroma descriptors and intensity to each compound. This initial research on the popular two cold-hardy varieties was a “screening”-type work aiming to find odor-active compounds. At this (screening) stage, using one panelist is sufficient to achieve the stated aims, i.e., to preliminarily characterize odor-active compounds. This information should be used for follow-up studies as a starting point for proper experimental design. Since ethanol was expected to be present in each sample, the intensity of ethanol was assigned as 50 on a 1 to 100 intensity scale. This process has been described in detail elsewhere [22,23].

### 2.6. Data Analysis

Odor-active compounds collected from wine headspace was tentatively identified by matching mass spectra to the NIST11 library, Wiley 6N library. All compounds with 80% spectral match or higher and above the 1000 peak area count threshold were considered for the analyses. Aroma descriptors from the panelist were compared to known aroma descriptors for additional verification. The matching of retention time indices was not appropriate in this case due to the GC columns of different polarity in-series configurations. The identification of compounds by the analysis of the pure standard was not performed, but the specific ions of a compound are provided in Table A1, when present in the chromatogram above the threshold.

Aroma extract dilution analysis (AEDA) was used to identify the most important compounds. From the aromagram, the odor dilution (OD) of each aroma event was multiplied by the measured intensity value resulting in the weighted intensity. This data was plotted with intensity (% full-scale) vs. time. Compounds with a higher OD were considered to be major contributors to the aroma profile of the wine.

Aroma descriptor intensity and OD were analyzed by principal components analysis (PCA) and cluster summary analysis using JMP Pro 12.0.1 (SAS, Cary, NC, USA). PCA is useful in summarizing all the odor-active compounds, detected by the human nose, in the wines among all conceivable linear combinations. A cluster summary analysis was also performed to determine the most representative aroma compound (i.e., the cluster variable with the largest squared correlation with its cluster component).

## 3. Results

Aroma events were simultaneously recorded using the sniff port by a trained human panelist during chromatographic analysis. A summary of the aroma events and the tentative identification by mass spectra is given in Table A1 in Appendix A. There were 57 unique aroma events detected by olfactometry and 32 odor-active compounds tentatively identified by mass spectrometry in Frontenac gris and Brianna wines. There were 35 and 34 aroma events recorded for Frontenac gris and Brianna wines, respectively. Aroma descriptors that were common between Frontenac gris and Brianna wines included “alcoholic, banana, body odor, butterscotch, cut grass, floral, fruity, garlic, honey, caramel, overripe fruit, rose, rotten eggs, solvent, strawberry”, and “tomato”. Aroma descriptors unique to Frontenac gris wines included “woody, carrots, cereal, mushroom, sweaty”, and “vinegar”. Aroma descriptors unique to Brianna wines included “barnyard, cotton candy, and mint.” The intensity of aromas (detailed in Materials and Methods section) in Frontenac gris and Brianna wines, according to harvesting parameters, is summarized in Table 3.

Seventeen aromas did not yield suitable (>80%) corresponding mass spectral matches and are labeled as “unknown”. This could indicate that the compound responsible for this aroma is not concentrated enough for the mass detector to respond and that the odor detection threshold for this compound was very low. The evidence that the human nose can be more sensitive than the chemical detector is consistent with the notion that simultaneous chemical and sensory analyses are useful for analyses of complex wine headspace. Wine headspace aroma is one of the first attributes experienced by consumers and wine enthusiasts.

### 3.1. Frontenac Gris White Wine Aroma Analysis by SPME-GC-MS-O

**Figure 3:**
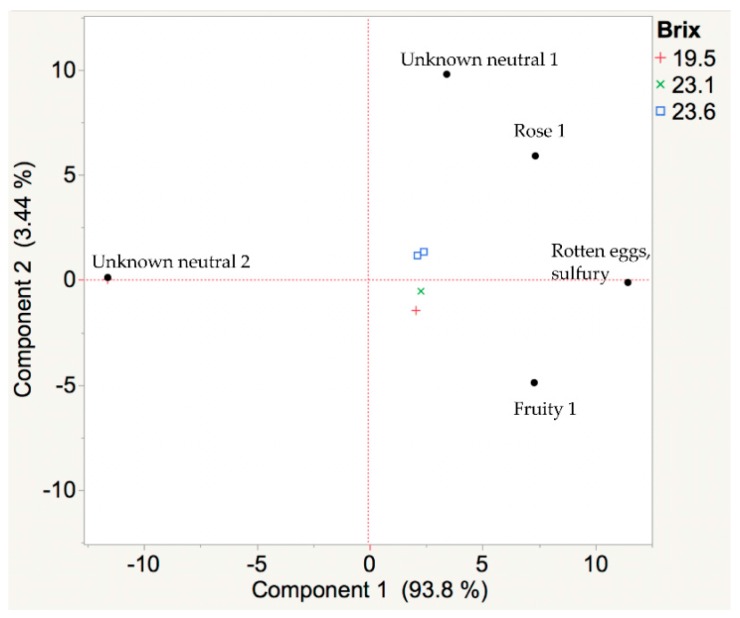
A principal components analysis (PCA) biplot of volatiles from the aroma extract dilution analysis of Frontenac gris wines made from berries harvested at three different ripening stages. Wines were made from Frontenac gris cold-hardy grapes harvested at 19.5, 23.1, and 23.6 °Brix. The juice was adjusted to 24 °Brix prior to fermentation. Wine headspace samples were collected by solid-phase microextraction (SPME) and analyzed with gas chromatography-mass spectrometry-olfactometry (GC-MS-O). Aroma descriptors were recorded by a trained human panelist. A shift of the aroma profile from “fruity 1”to “rotten eggs, sulfury” to “rose 1” was observed. Over 98% of the variation in harvest time is correlated with key odor-active compounds.

White wines from Frontenac gris grapes had 35 recorded aroma events across all samples. Aromas of “honey, caramel, butterscotch” and “strawberry, honey” had no variation in odor dilution (OD, defined in Methods) and were not used in the multivariate analysis. The aromas with the highest intensity in the Frontenac gris wines were “banana”, “fruity 2”, “honey”, and “unknown neutral 1”. Cluster summary analysis of OD showed that “rotten eggs, sulfury” and “unknown neutral 1” were the most representative aromas in these Frontenac gris wine. A “rotten eggs” smell in wine is considered a wine fault due to the winemaking process and therefore not considered a characteristic aroma of the grape. A chromatographic peak was not present at the corresponding retention time for “unknown neutral 1”. As pH and sugar accumulation in the berry increased, key odor-active compounds in these Frontenac gris wines developed from “unknown neutral 2” and “fruity 1” to “rose 1” (Figure 3). These correspond to mass spectral matches of “unknown neutral 2” to decanoic acid (CAS 334-48-5) and “fruity 1” to ethyl methylbutyrate (CAS 7452-79-1). A suitable mass spectral match was not found for the identification of “rose 1.” An open source aroma database [7] lists the percepts of “rancid, fat” for decanoic acid and “apple, characteristic of Golden delicious” for ethyl methylbutyrate. In the Flavornet database, 16 different compounds are listed with the aroma descriptor “rose”.

### 3.2. Brianna White Wine Aroma Analysis by SPME-GC-MS-O

**Figure 4:**
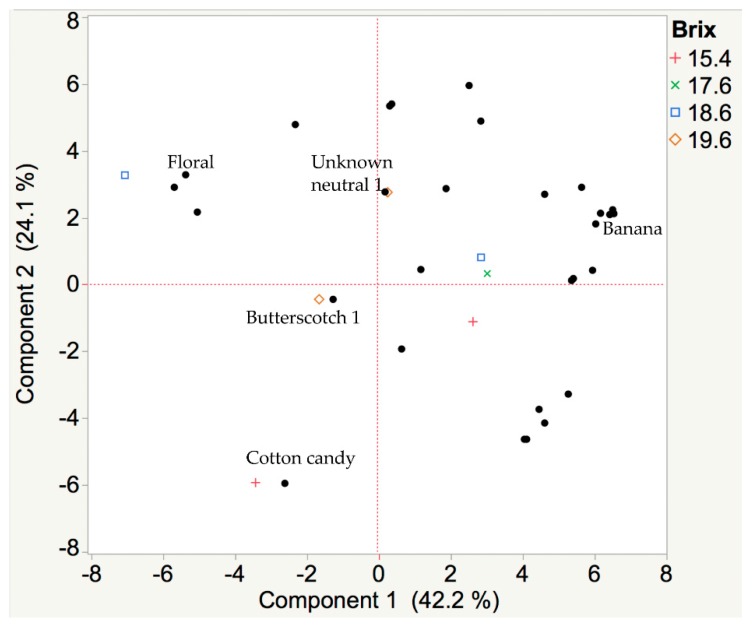
A PCA biplot of aromas from Brianna wines made from berries harvested at four different ripening stages. Wines were made from Brianna cold-hardy grapes harvested at 15.4, 17.6, 18.6, and 19.6 °Brix. The juice was adjusted to 20 °Brix for all time points prior to fermentation. Wine headspace samples were collected by SPME and analyzed with GC-MS-O. Aroma descriptors were recorded by a trained human panelist. A shift of the aroma profile from “cotton candy” to “banana” to “floral” to “butterscotch” was observed. Over 68% of the variation in harvest time is correlated with key odor-active compounds.

White wine from Brianna grapes had 34 recorded aroma events across all samples. The “rotten eggs” aroma had no variation in OD and was not used in the multivariate analysis similarly to Frontenac gris. The most intense aromas in these Brianna wines were “overripe fruit 2”, “floral”, and “unknown neutral 5”. The most representative aromas, as indicated by cluster analysis, in these Brianna wines were “banana”, “floral”, “honey, caramel”, “butterscotch 1”, “tomato 1”, and “overripe fruit 2”. Corresponding compounds from mass spectral searches are isoamyl acetate (banana, CAS 123-92-2), ethyl isobutyrate (“honey, caramel”, CAS 97-62-1), and isoamyl alcohol (“overripe fruit 2”, CAS 123-51-3). A suitable mass spectral match was not found for the “floral” aroma compound. A chromatographic peak was not recorded corresponding to “butterscotch 1”, although the database lists methyl vanillate [7] as a source of this aroma. Two mass spectral matches were identified for “tomato 1”: diphenylmethane (“green”, CAS 101-81-5) [24] and isobutyl decanoate (“fermented”, CAS 30673-38-2) [24]. The “floral” aroma is associated with 48 different compounds [7]. As pH and sugar accumulation in these Brianna berries increased, key odor-active compounds for each harvest changed (Figure 4). When harvested at the lowest sugar content and pH, the wines had a “cotton candy” (ethyl decanoate, CAS 110-38-3) and “floral” aroma. From 17.6 to 18.6 °Brix, aromas changed from “banana” to “butterscotch.” At the highest sugar and pH, the key aromas in the Brianna wines were “honey, caramel” and “unknown neutral 1” (isobutyl alcohol, CAS 78-83-1). This change in aromas over Brianna berry ripening is shown in Figure 4.

## 4. Discussion

SPME has been used to quantify volatile by-products in industrial ethanol [25], volatile cogeners in food-grade ethanol [23], and volatile odor-active compounds in cold-hardy wines made from Marquette and Frontenac [22] and even used to characterize street drug aromas [26,27,28]. Odor-active compounds in wine headspace must be extracted quickly and efficiently in order to minimize the effects of oxidation on the wine aroma profile. In this research, a SPME 50/30 µm divinylbenzene (DVB)/Carboxen/polydimethylsiloxane (PDMS) coating was suitable for extraction of a wide variety of aroma volatiles including alcohols, esters, aldehydes and ketones, phenolics, and acids. Ethanol being the most prevalent in headspace did not outcompete volatile aromas for SPME sorption sites.

Simultaneous sensory and chemical analyses of white wine aroma was facilitated by the use of GC-MS-O. The advantage of using olfactometry (O) simultaneously with chemical detection is the ability to focus on selected fewer aroma-causing compounds present in a very complex mixture of the wine headspace matrix. A sole focus on chemical analyses can preclude finding the aroma-defining volatile compounds in wine.

Grape sugar content (°Brix) varies depending on the species, variety, maturity (ripening), and health of the fruit [10]. Cultivars of European *Vitis vinifera* generally accumulate sugar at a concentration of 20% or more at maturity [29]. The cold-hardy cultivars Brianna and Frontenac gris pedigree includes *V. riparia*, *V. labrusca*, and *V. vinifera* [30,31]. Brianna, in particular, is often harvested at a lower °Brix to avoid “foxy” flavors. Sugar is added (chaptalization) to the juice to develop the 10–12% alcohol content typical of most still (non-sparkling) table wines [32]. The effects of sugar content and ethanol concentrations on the sensory attributes of young and aged sweet wines is found elsewhere [33,34]. However, there are few intervention options for enhancing the desired aromas. Thus, wine cold-hardy white wines produced from European/native N. American cultivars such as Brianna and Frontenac gris need to be “farmed for flavor.” This means that growers should consider an optimal flavor profile as a harvest parameter, in addition to the °Brix, pH, and TA.

Grapes produce few aldehydes significant in varietal aromas. This may result from their reduction to alcohols during primary fermentation. Of the aldehydes not metabolized during primary fermentation, C-6 aldehydes appear to be the most noteworthy [35]. These aldehydes are responsible for the grassy to herbaceous odor associated with certain grape varieties or with wines made from immature grapes. They appear to be formed during crushing by the enzymatic oxidation of grape lipids [4]. Most aldehydes found in wine are created during processing or fermentation or are extracted from oak cooperage [32].

Likewise, few ketones are found in grapes. The norisoprenoid ketones (i.e., beta-damascenone, alpha-ionone, and beta-ionone) are persistent throughout fermentation [32]. The “apple, rose, honey” aroma of beta-damascenone [7] and low odor threshold [24] imply that it is important in the aroma of several grape varieties including “Chardonnay” [36] and “Riesling” [37]. The “seaweed, violet, flower, raspberry” aroma of beta-ionone [7], along with beta-damascenone, are important in the aroma of several red grape varieties [38]. Other ketones that are generated by fungal metabolism or produced during fermentation and acetals produced during aging and distillation will not be discussed in this research.

Of all the aromatic constituents of wine, esters are the most abundant. Most of these esters are found only in trace amounts and have either low volatility or non-distinct odors, and their importance to wine fragrance is often discounted. The more common esters such as acetate esters are derived from acetic acid and fusel alcohols, and the ethyl esters are formed between ethanol and fatty acids or nonvolatile, fixed organic acids. The fruity aromas are important in the aroma profile of young white wines [39]; however, the esters to the aromas of red wines is less understood.

Terpenes are an important group of aromatic compounds characterizing the aromas of “flower and lavender” (linalool), “rose and geranium” (geraniol), “sweet” (nerol), “oil, anise, mint” (alpha-terpineol), and “hyacinth” (hotrienol) [7]. Terpenes are responsible for the fragrance of herb-flavored wines such as vermouth and fruit-flavored wines. In addition, terpenes also characterize some wine grape cultivars, most notably the “Muscat” and “Riesling” families [40].

Pyrazines are important to the characteristic varietal aromas of several cultivars [41]. Ethyl 3-mercaptopropionate is an important compound suspected to be the “foxy” odor of some *V*. *labrusca* varieties [42]. Most thiols generate off-odors, and only a few contribute to the characteristic varietal aroma of wine grape cultivars. These are 4-mercapto-4-methylpentan-2-ol (“floral, lemon grapefruit”) [24] and 3-mercaptohexan-1-ol (“grapefruit”) [43]. Both compounds are important in the varietal character of “Sauvignon Blanc” [43]. A key aroma important in “Scheurebe” is 4-mercapto-4-methylpentan-2-ol [44].

Despite the information available on volatile wine odor-active compounds and their sensory perceptions, experienced tasters are not always able to determine the grape variety (Vinifera), even when 100% of the wine is made from that cultivar [45]. A review of wine aroma in grapes is provided elsewhere [46]. The question remains if these new cold-hardy cultivars produce a distinct varietal aroma in white wines. This research adds a valuable initial report on white wine aromas from Brianna and Frontenac gris grapes. To date, the only other published research on cold-hardy wine aromas pertains to red wines [47,48,49,50] and white wines [51]. Therefore, this research serves as a starting point for determining the odor-active compounds in Brianna and Frontenac gris cold-hardy wines. At this (screening) stage, using one panelist achieved the stated aims, i.e., preliminarily characterized odor active compounds. This information should be used for follow-up studies as a starting point for proper experimental design. This relatively low number of publications on cold-hardy wine varieties is significant compared with active research in Vinifera [52,53,54,55,56,57,58,59,60,61,62].

Results (obtained with GC-MS-O approach) from this research could be used to inform cold-hardy grape growers on “farming for flavor.” A shift of the aroma profile from “fruity 1” to “rotten eggs, sulfury” to “rose 1” was observed in wines made from Frontenac gris harvested at 19.5, 23.1, and 23.6 °Brix, respectively (Figure 3). The must was not submitted to cold-settling and might be a major reason for the “sulfury, rotten egg” odors found in the research wines. In addition, a shift of the aroma profile from “cotton candy” to “banana” to “floral” to “butterscotch” was observed in wines that were made from Brianna grapes harvested at 15.4, 17.6, 18.6, and 19.6 °Brix, respectively (Figure 4).

**Figure 5:**
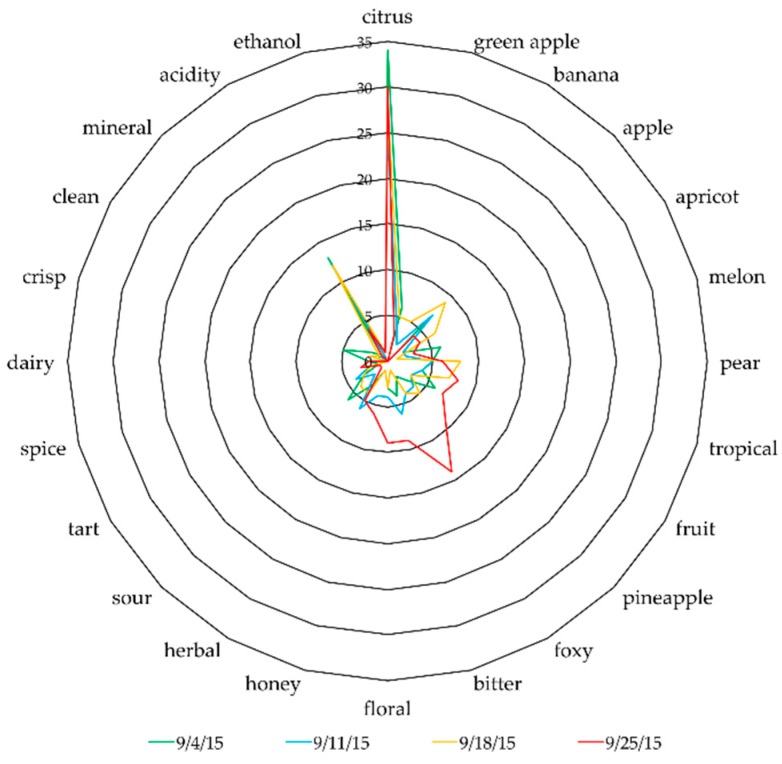
A spiderplot indicating a shift of flavor and aroma descriptors of Brianna wines, that were made from grapes harvested from 4 September to 25 September, from the wine tasting panels generated by lay audiences at conferences for wine industry professionals. A shift of flavor and aroma descriptors is associated with the increase in °Brix and appearance of “foxy”, a negative characteristic associated with *V. labrusca*-based wines at the latest harvest date.

Similar shifts of actual flavor and aroma of wines made from Brianna were also observed during tasting sessions at conferences for wine industry professionals. Results from the lay audiences’ flavor and aroma descriptors (Figure 5) also indicate a shift with harvest date and associated °Brix. The most obvious change at the late harvest date is the use of the term “foxy”, a negative characteristic associated with *V. labrusca*-based wines. There was also a decrease in the use of “acidity,” although “citrus” was still mentioned. Additional flavor descriptors that had a higher incidence in the late harvested wine included “bitter”, “floral”, and “pineapple”. The lay audiences’ perceptions of the Brianna wine detected some of the “sulfur”, “dirty”, “musty” aromas but at a very low incidence.

This research will help support the sustainable development of cold-hardy grape growing and the winemaking industry in Midwest U.S by providing a baseline for viticultural and wine-making practices. The next logical step would be to relate aroma-active compounds with sensory attributes by means of pattern recognition techniques that use multivariate statistical tests such, as principal component analysis, cluster analysis, or even partial least square (PLS) algorithms as previously described [63,64]. It is also possible to use the volatile data obtained by GC to construct odorant series with a given odor activity value for comparison purposes with sensorial data as in References [26,27,28,65,66,67].

More research is warranted on the aromas of white wines produced from cold-hardy cultivars. Several recommendations could be made including repeated studies involving a greater number of growing seasons and eventually developing consistent regional wine styles. This could include linking the sensory characteristics such as color, body and mouthfeel [68], and aroma.

## 5. Conclusions

This is the first report of white wine aromas from cold-hardy Brianna and Frontenac gris by GC-MS-O. Findings from this research support the hypothesis that aroma profiles of Brianna and Frontenac gris wines can be influenced by harvesting the grapes at different stages of ripening. Evaluation of the respective cultivar wines from different harvest dates but the same alcohol content allowed the detection of over 30 odor-active compounds in the wine headspace for both Brianna and Frontenac gris. The particular wine aroma profile changed depending on the time of harvest and grape maturity. Aromas in Brianna wines developed from “cotton candy” and “floral” to “banana” and “butterscotch” and then finally to “honey”, “caramel”, and an “unknown neutral” aroma. Over 68% of the variation in harvest time was correlated with key odor-active compounds. Aromas in Frontenac gris wines changed from an “unknown neutral” aroma to “fruity” to “rose”. Over 98% of the variation in harvest time was correlated with key odor-active compounds. Wine tasting data generated by wine industry professionals at conferences showed a shift in flavor and aroma descriptors for Brianna wines. The shift of flavor and aroma descriptors is associated with the increase in °Brix and “foxy,” a negative characteristic associated with *V. labrusca*-based wines at the latest harvest dates. This research provides both positive and negative aroma characteristics associated with increased ripeness and will help support the sustainable development of cold-hardy grape growing and the winemaking industry in Midwest U.S by providing a baseline for viticultural and wine-making practices.

## Figures and Tables

**Table 1:** Economic impact of the U.S. Midwest (cold climate) wine industry [1].

State	Economic Impact ^1^	Jobs	Wages ^1^	Vineyard Activity ^1^	Winery Activity ^1^	Tourism ^1^	Other ^1,2^
North Dakota	$135	2340	$57.3	$0.00680	$7.09	$0.245	$127
South Dakota	$180	2690	$62.4	$0.0719	$25.7	$1.69	$153
Iowa	$573	8760	$197	$1.10	$110	$29.0	$433
Minnesota	$979	15,400	$408	$1.22	$83.5	$21.3	$873
Wisconsin	$1320	20,700	$519	$1.12	$146	$39.7	$1130
Michigan	$1890	25,800	$710	$7.77	$325	$149	$1410
Illinois	$2480	34,800	$1060	$1.82	$247	$222	$2010
Totals	$7550	11,0000	$3010	$13.1	$944	$463	$6130

^1^ Millions of U.S. Dollars (USD); ^2^ includes wholesale, retail, associations, research, and education.

**Table 2:** Brianna and Frontenac gris grapes’ characteristics at harvest.

Cultivar	Harvest Date	Berry °Brix	Berry pH
Frontenac gris	24 September 2015	19.5	3.00
Frontenac gris	1 October 2015	23.1	3.06
Frontenac gris	9 October 2015	23.6	3.18
Brianna	4 September 2015	15.4	3.09
Brianna	11 September 2015	17.6	3.19
Brianna	18 September 2015	18.6	3.29
Brianna	25 September 2015	19.6	3.45

**Table 3:** Summary of the ranked weighted intensity of aromas (recorded by olfactometry) in wine made from Frontenac gris and Brianna grapes harvested at different stages of ripening. All juice was brought to 24 °Brix for Frontenac gris and 20 °Brix for Brianna prior to fermentation to ensure similar alcohol content in the wines from the different time points.

Cultivar	Berry °Brix	Berry pH	Aroma Descriptors (Weighted Intensity ^1^)
Frontenac gris	19.5	3.00	unknown pleasant (19), floral/fruity (11), floral (5), overripe (3), butterscotch (2), tomato (1), unknown pleasant 1 (0), unknown neutral 2 (0)
Frontenac gris	23.1	3.06	honey/caramel/butterscotch (431), fruity (419), cut grass/fruity (417), alcoholic (391), banana (382), body odor (359), fruity 1 (345), solvent (337), unknown pleasant (324), rose 2 (321), garlic (207), carrots/woody (178), cereal (152), honey (122), vinegar (57), woody 1 (55)
Frontenac gris	23.6	3.18	strawberry (524), strawberry/honey (395), sweaty (384), fruity 2 (244), match/sulfury (183), rose 1 (132), fecal (117), woody 2 (102), rotten eggs/sulfury (78), mushroom (63), unknown neutral 1 (5)
Brianna	15.4	3.09	rose (158), body odor (123), barnyard (122), butterscotch 2 (115), unknown pleasant (111), unknown neutral 2 (98), matchstick (92), mint (67), cotton candy (13)
Brianna	17.6	3.19	alcoholic (420), overripe fruit 2 (373), rotten eggs (106)
Brianna	18.6	3.29	strawberry 2 (579), fruity 3 (506), cut grass (500), floral/fruity (472), honey/caramel (468), banana (467), overripe fruit 1 (455), solvent (425), strawberry 1 (382), unknown neutral 3 (363), fruity 2 (316), garlic (239), unknown pleasant (193), fruity 1 (21), floral (5)
Brianna	19.6	3.45	tomato 2 (196), unknown neutral 4 (194), unknown neutral 5 (180), tomato 1 (179), unknown neutral 1 (59), fruity 4 (45), butterscotch 1 (3)

^1^ Defined in the Materials and Methods (Section 2.6).

## Appendix A

**Table A1:** Summary of identified aromas and associated compounds in the headspace of wines made from Brianna and Frontenac gris cold-hardy grapes. Percent match to NIST 11 mass spectral library was equal or greater than 80%.

Event Number	Aroma Descriptor	Weighted Intensity	Retention time (min)	Aroma Event Width (min)	OD *	Mass Spectral Library Identification	Chemical Abstracts Service Number	Significant Ions (Number of Ions Listed: Ions Listed in the Order of Intensity)
Variety: Brianna; Harvest Date: 4 September 2015; Sample Number: 1								
1	Rotten eggs	79	2.54	0.03	32	Not detected		
2	Alcoholic	307	3.2	0.19	32	Ethanol	64-17-5	5: 43 45 60 42 44
3	Butterscotch 2	115	4.81	0.08	32	Ethyl lactate	97-64-3	7: 45 75 43 46 47 61 103
4	Body odor	118	5.31	0.08	32	Isobutyl alcohol	78-83-1	11: 41 42 39 74 57 59 73 40 37 58 52
5	Honey, caramel	256	5.75	0.11	32	Ethyl isobutyrate	97-62-1	10: 71 89 60 41 101 73 102 90 59 88
6	Floral, fruity	312	6.91	0.08	32	Ethyl butyrate	105-54-4	2: 107 108
7	Solvent	302	7.2	0.07	32	Unknown		
8	Over ripe fruit 1	283	7.56	0.09	32	Isoamyl alcohol	123-51-3	15: 60 73 41 87 43 55 42 57 39 61 69 59 99 50 58
9	Over ripe fruit 2	311	8	0.16	32	Isoamyl alcohol	123-51-3	0
10	Fruity 2	177	8.27	0.08	32	Ethyl isovalerate	108-64-5	0
11	Banana	273	9.04	0.08	32	Isoamyl acetate	123-92-2	20: 91 92 122 65 51 39 77 93 63 103 104 50 62 52 64 79 66 38 75 102
12	Unknown pleasant	111	11.32	0.09	32	Unknown		
13	Fruity 3	369	12.22	0.1	32	Ethyl hexanoate	123-66-0	4: 74 71 87 59
14	Garlic	63	12.83	0.11	32	Not detected		
15	Unknown neutral 2	98	15.18	0.09	32	Unknown		
16	Rose	158	15.91	0.16	32	Unknown		
17	Matchstick	92	16.7	0.08	32	Unknown		
18	Cut grass	333	17.06	0.2	32	Ethyl octanoate	106-32-1	17: 108 107 150 77 43 79 109 80 90 51 78 53 50 39 89 62 105
19	Barnyard	122	18.43	0.16	32	4-methylphenyl acetate	140-39-6	10: 43 71 116 88 41 89 42 57 44 70
20	Mint	67	19.3	0.07	32	Methyl salicylate	119-36-8	8: 57 85 212 112 83 97 141 113
21	Unknown neutral 3	264	19.97	0.07	32	Phenethyl alcohol	60-12-8	20: 88 101 155 73 157 70 43 55 41 60 61 89 69 57 71 115 143 83 42 85
22	Strawberry 1	309	21.52	0.35	32	Ethyl decanoate	110-38-3	17: 99 117 56 43 71 60 55 57 41 73 39 100 87 69 116 101 118
23	Strawberry 2	406	22.09	0.08	32	Octanoic acid	124-07-2	14: 71 88 43 73 60 89 101 70 61 42 39 116 55 90
**Variety: Brianna; Harvest Date: 04 September 2015; Sample Number: 2**								
1	Rotten eggs	70	2.54	0.03	32	Not detected		
2	Alcoholic	383	3.2	0.19	32	Ethanol	64-17-5	15: 45 46 43 47 42 41 44 40 33 48 77 49 39 78 34
3	Butterscotch 2	106	4.81	0.08	32	Unknown		
4	Body odor	123	5.31	0.45	32	Isobutyl alcohol	78-83-1	18: 43 41 42 33 39 74 55 56 57 40 59 44 53 37 50 54 49 52
5	Honey, caramel	255	5.67	1.38	32	Ethyl isobutyrate	97-62-1	5: 71 116 73 88 89
6	Solvent	320	7.25	0.01	32	Unknown		
7	Over ripe fruit 1	2	7.52	0.04	1	Isoamyl alcohol	123-51-3	20: 55 70 42 43 39 45 69 71 46 44 40 38 51 50 47 60 37 67 52 73
8	Over ripe fruit 2	2	7.99	0.06	1	Isoamyl alcohol	123-51-3	20: 55 70 42 43 39 45 69 71 46 44 40 38 51 50 47 60 37 67 52 73
9	Over ripe fruit 2	1	8.23	0.03	1	Ethyl isovalerate	108-64-5	6: 88 85 60 61 115 87
10	Banana	2	9.04	0.03	1	Isoamyl acetate	123-92-2	19: 43 70 55 87 61 42 41 73 69 39 44 58 88 57 53 85 54 115 40
11	Unknown pleasant	3	11.32	0.04	2	Unknown		
12	Fruity 3	360	12.25	0.03	32	Ethyl hexanoate	123-66-0	20: 88 99 43 101 60 70 71 73 61 41 55 42 115 45 39 87 69 117 89 100
13	Garlic	0	12.82	0.03	1	Not detected		
14	Unknown neutral 2	34	15.17	0.08	32	Unknown		
15	Rose	1	15.94	0.03	1	Unknown		
16	Matchstick	2	16.69	0.02	2	Unknown		
17	Cut grass	7	17.02	0.08	2	Ethyl octanoate	106-32-1	20: 88 101 127 57 73 70 60 55 41 61 43 129 115 89 42 69 45 83 143 39
18	Barnyard	2	18.41	0.09	2	Unknown		
19	Mint	1	19.38	0.04	1	Unknown		
20	Unknown neutral 3	322	20	0.05	32	Phenethyl alcohol	60-12-8	20: 91 92 122 65 39 51 77 63 93 78 89 103 123 104 50 62 90 52 64 66
21	Cotton candy	13	21.42	0.07	2	Ethyl decanoate	110-38-3	20: 88 101 155 73 157 70 43 55 41 60 61 89 69 57 71 115 143 83 42 85
22	Strawberry 2	3	21.98	0.24	1	Octanoic acid	124-07-2	20: 60 73 43 101 41 55 85 84 87 69 115 61 39 45 57 74 83 67 97 102
23	Unknown neutral 4	1	24.45	0.05	1	Unknown		
24	Unknown neutral 4	2	24.74	0.04	1	Unknown		
25	Unknown neutral 5	1	25.2	0.05	1	Decanoic acid	334-48-5	20: 73 60 129 71 57 41 55 43 69 87 115 83 61 84 39 143 74 112 56 42
**Variety: Brianna; Harvest Time: 11 September 2015; Sample Number: 1**								
1	Rotten eggs	106	2.54	0.03	32	Not detected		
2	Alcoholic	420	3.2	0.19	32	Ethanol	64-17-5	20: 45 46 43 42 47 41 44 33 40 48 77 49 39 61 78 34 53 55 38 165
3	Butterscotch 2	71	4.81	0.08	32	Ethyl lactate	97-64-3	2: 45 75
4	Body odor	95	5.31	0.08	32	Isobutyl alcohol	78-83-1	20: 43 41 42 33 39 74 55 56 57 40 59 38 73 44 53 37 50 51 72 34
5	Honey, caramel	355	5.75	0.11	32	Ethyl isobutyrate	97-62-1	10: 43 71 116 41 88 73 89 42 72 101
6	Floral, fruity	361	6.91	0.08	32	Ethyl butyrate	105-54-4	17: 71 88 43 41 60 89 42 101 39 61 116 72 117 90 40 38 47
7	Solvent	337	7.2	0.07	32	Unknown		
8	Over ripe fruit 1	345	7.56	0.09	32	Isoamyl alcohol	123-51-3	20: 55 70 41 42 43 45 69 71 46 44 40 38 54 60 37 67 35 52 62 63
9	Over ripe fruit 2	373	8	0.16	32	Isoamyl alcohol	123-51-3	20: 55 70 41 42 43 45 69 71 46 44 40 38 54 60 37 67 35 52 62 63
10	Fruity 2	91	8.27	0.08	32	Ethyl isovalerate	108-64-5	9: 88 85 60 61 87 115 59 103 86
11	Banana	308	9.04	0.08	32	Isoamyl acetate	123-92-2	19: 43 70 55 87 61 41 42 73 69 39 71 56 88 44 58 57 85 53 40
12	Unknown pleasant	55	11.32	0.09	32	Unknown		
13	Fruity 3	394	12.22	0.1	32	Ethyl hexanoate	123-66-0	20: 88 99 43 101 60 70 71 73 61 41 55 42 115 45 87 39 117 69 89 102
14	Garlic	40	12.83	0.11	32	Not detected		
15	Unknown neutral 2	57	15.18	0.09	32	Unknown		
16	Rose	123	15.91	0.16	32	Unknown		
17	Matchstick	39	16.7	0.08	32	Unknown		
18	Cut grass	423	17.06	0.2	32	Ethyl octanoate	106-32-1	20: 88 101 127 57 73 70 55 60 41 61 43 129 115 42 89 69 45 83 143 39
19	Barnyard	40	18.43	0.16	32	Not detected		
20	Mint	49	19.3	0.07	32	Not detected		
21	Unknown neutral 3	345	19.97	0.07	32	Phenethyl alcohol	60-12-8	20: 91 92 122 65 39 63 77 51 93 78 89 103 123 104 50 62 52 90 64 41
22	Strawberry 1	307	21.49	0.38	32	Ethyl decanoate	110-38-3	20: 88 101 155 157 73 70 55 41 43 60 61 69 89 115 57 71 143 83 42 45
23	Strawberry 2	498	22.01	0.16	32	Octanoic acid	124-07-2	20: 60 73 43 55 41 101 85 84 87 69 61 39 115 45 42 57 56 74 83 82
24	Tomato 1	63	24.32	0.02	32	Not detected		
25	Unknown neutral 4	162	24.78	0.02	32	Unknown		
26	Unknown neutral 5	94	25.27	0.02	32	Decanoic acid	334-48-5	19: 73 60 129 41 55 57 43 71 69 87 115 83 61 172 42 84 143 39 56
**Variety: Brianna; Harvest Time: 11 September 2015; Sample Number: 2**								
1	Rotten eggs	54	2.54	0.03	32	Not detected		
2	Alcoholic	420	3.2	0.19	32	Ethanol	64-17-5	17: 45 46 43 42 47 41 44 33 40 48 77 49 39 61 91 78 95
3	Butterscotch 2	71	4.81	0.08	32	Unknown		
4	Body odor	95	5.31	0.08	32	Ethyl propionate	105-37-3	
5	Honey, caramel	355	5.75	0.11	32	Ethyl isobutyrate	97-62-1	6: 71 116 88 33 73 117
6	Floral, fruity	361	6.91	0.08	32	Unknown		
7	Solvent	337	7.2	0.07	32	Ethyl butyrate	105-54-4	14: 71 88 43 73 41 89 101 70 61 72 116 57 69 37
8	Over ripe fruit 1	345	7.56	0.09	32	Unknown		
9	Over ripe fruit 2	373	8	0.16	32	Isoamyl alcohol	123-51-3	10: 70 42 43 39 44 46 51 59 37 49
10	Fruity 2	91	8.27	0.08	32	Not detected		
11	Banana	308	9.04	0.08	32	Isoamyl acetate	123-92-2	20: 43 70 55 87 61 41 42 73 69 39 88 58 56 44 85 57 53 45 54 40
12	Unknown pleasant	55	11.32	0.09	32	Unknown		
13	Fruity 3	394	12.22	0.1	32	Unknown		
14	Garlic	40	12.83	0.11	32	Not detected		
15	Unknown neutral 2	57	15.18	0.09	32	Ethyl heptanoate	106-30-9	10: 88 113 101 84 87 74 69 83 89 102
16	Rose	123	15.91	0.16	32	Unknown		
17	Matchstick	39	16.7	0.08	32	Unknown		
18	Cut grass	423	17.06	0.2	32	Ethyl octanoate	106-32-1	20: 88 101 127 57 73 70 55 60 41 61 43 129 115 89 42 69 143 83 45 39
19	Barnyard	40	18.43	0.16	32	Unknown		
20	Mint	49	19.3	0.07	32	Unknown		
21	Unknown neutral 3	345	19.97	0.07	32	Unknown		
22	Strawberry 1	307	21.49	0.38	32	Unknown		
23	Strawberry 2	498	22.01	0.16	32	Octanoic acid	124-07-2	20: 60 73 43 55 41 101 85 84 87 69 115 61 45 39 42 57 56 74 83 82
24	Tomato 1	63	24.32	0.02	32	Diphenylmethane	101-81-5	13: 167 168 165 152 169 166 76 63 141 128 164 50 78
25	Unknown neutral 4	162	24.78	0.02	32	Unknown		
26	Unknown neutral 5	94	25.27	0.02	32	Decanoic acid	334-48-5	19: 73 60 129 55 41 57 71 43 87 69 83 115 61 84 39 74 42 143 70
**Variety: Brianna; Harvest Time: 18 September 2015; Sample Number: 1**								
1	Rotten eggs	87	2.54	0.03	32	Not detected		
2	Alcoholic	411	3.2	0.19	32	Ethanol	64-17-5	17: 45 46 43 42 47 41 44 33 40 48 77 49 91 55 78 51 92
3	Butterscotch 2	109	4.81	0.08	32	Unknown		
4	Body odor	42	5.31	0.08	32	Isobutyl alcohol	78-83-1	13: 43 41 42 39 74 55 56 57 38 53 44 73 37
5	Honey, caramel	468	5.75	0.11	32	Ethyl isobutyrate	97-62-1	10: 43 71 88 41 116 55 73 42 39 72
6	Floral, fruity	472	6.91	0.08	32	Ethyl butyrate	105-54-4	16: 71 88 43 73 60 41 89 101 42 70 61 39 55 40 38 62
7	Solvent	425	7.2	0.07	32	Unknown		
8	Over ripe fruit 1	455	7.56	0.09	32	Unknown		
9	Over ripe fruit 2	360	8	0.16	32	Unknown		
10	Fruity 2	117	8.27	0.08	32	Isoamyl alcohol	123-51-3	13: 55 70 42 43 39 45 46 44 53 40 73 66 62
11	Banana	467	9.04	0.08	32	Isoamyl acetate	123-92-2	20: 43 70 55 87 61 41 42 73 69 39 71 44 88 56 58 57 85 53 45 54
12	Unknown pleasant	71	11.32	0.09	32	Unknown		
13	Fruity 3	506	12.22	0.1	32	Ethyl hexanoate	123-66-0	20: 88 99 43 101 60 70 71 73 41 61 55 42 115 45 39 87 69 117 89 100
14	Garlic	106	12.83	0.11	32	Not detected		
15	Unknown neutral 2	34	15.18	0.09	32	Rose oxide	16409-43	9: 139 69 83 154 140 84 85 53 77
16	Unknown unpleasant	71	15.69	0.07	32	Unknown		
17	Rose	129	15.91	0.16	32	Unknown		
18	Matchstick	41	16.7	0.08	32	Unknown		
19	Cut grass	500	17.06	0.2	32	Ethyl octanoate	106-32-1	20: 88 101 127 57 73 70 55 60 41 61 43 129 115 42 89 69 143 45 83 39
20	Barnyard	11	18.43	0.16	32	Unknown		
21	Mint	26	19.3	0.07	32	Methyl salicylate	119-36-8	5: 120 152 92 65 149
22	Unknown neutral 3	363	19.97	0.07	32	Phenethyl alcohol	60-12-8	20: 91 92 122 65 77 93 51 39 63 78 103 104 50 62 90 52 79 41 53 75
23	Strawberry 1	382	21.49	0.38	32	Ethyl decanoate	110-38-3	20: 88 101 155 157 73 70 55 41 43 60 61 69 89 57 115 71 143 83 42 85
24	Strawberry 2	579	22.01	0.16	32	Octanoic acid	124-07-2	20: 60 73 43 101 55 41 85 84 87 69 115 39 61 45 42 57 56 74 83 82
25	Tomato 1	73	24.32	0.02	32	Unknown		
26	Unknown neutral 4	154	24.78	0.02	32	Unknown		
27	Unknown neutral 5	94	25.27	0.02	32	Decanoic acid	334-48-5	20: 73 60 129 55 57 41 71 43 69 87 83 115 110 61 84 112 74 56 70 53
**Variety: Brianna; Harvest Time: 18 September 2015; Sample Number: 2**								
1	Rotten eggs	46	2.54	0.03	32	Not detected		
2	Alcoholic	4	3.2	0.19	1	Ethanol	64-17-5	18: 45 46 43 42 47 41 44 33 40 48 77 49 39 55 91 84 97 104
3	Fruity 1	21	4.2	0.38	32	Not detected		
4	Butterscotch 2	18	4.81	0.08	32	Ethyl lactate	97-64-3	2: 45 75
5	Body odor	16	5.31	0.08	32	Isobutyl alcohol	78-83-1	12: 43 41 42 39 74 56 57 40 53 38 44 37
6	Honey, caramel	6	6.1	-0.24	1	Not detected		
7	Floral	5	7.35	-0.36	1	Not detected		
8	Over ripe fruit 1	2	7.65	-0.38	1	Not detected		
9	Over ripe fruit 2	6	7.82	0.28	1	Isoamyl alcohol	123-51-3	10: 70 41 39 45 53 38 58 50 72 87
10	Fruity 2	316	8.45	-0.1	32	Not detected		
11	Banana	6	9.4	-0.28	1	Isoamyl acetate	123-92-2	20: 43 70 55 87 61 41 42 73 69 39 71 58 56 44 88 57 53 85 45 40
12	Unknown pleasant	0	11.32	0.09	1	Unknown		
13	Garlic	16	13.08	-0.14	8	Not detected		
14	Garlic	239	13	0.07	32	Not detected		
15	Unknown unpleasant	193	15.65	0.15	32	2-Nonanone	821-55-6	14: 58 43 71 59 57 142 127 85 82 95 113 53 72 54
16	Rose	1	16.43	-0.36	1	Unknown		
17	Cut grass	6	17.09	0.17	1	Unknown		
18	Barnyard	1	18.43	0.16	1	Not detected		
19	Mint	28	19.3	0.07	32	Not detected		
20	Fruity 4	28	19.71	0.01	32	Unknown		
21	Unknown neutral 3	5	20.05	-0.01	1	Phenethyl alcohol	60-12-8	20: 91 92 122 65 77 39 51 78 89 103 123 104 50 62 52 64 38 79 75 120
22	Strawberry 1	320	21.5	0.37	32	Ethyl decanoate	110-38-3	20: 88 101 155 157 73 70 55 41 43 60 61 69 89 115 57 71 143 83 42 85
23	Strawberry 2	422	22.05	0.12	32	Octanoic acid	124-07-2	20: 60 73 55 43 101 41 85 84 87 69 115 61 39 45 57 56 74 83 82 53
24	Tomato 1	79	24.32	0.02	32	Not detected		
25	Tomato 2	91	24.78	0.02	32	Unknown		
26	Unknown neutral 5	146	25.27	0.02	32	Unknown		
**Variety: Brianna; Harvest Time: 25 September 2015; Sample Number: 1**								
1	Rotten eggs	48	2.54	0.03	32	Not detected		
2	Alcoholic	293	3.2	0.19	32	Ethanol	64-17-5	
3	Butterscotch 2	67	4.81	0.08	32	Unknown		
4	Unknown neutral 1	59	5.31	0.08	32	Isobutyl alcohol	78-83-1	13: 43 33 41 42 39 74 55 56 57 53 75 49 54
5	Honey, caramel	18	5.75	0.11	4	Ethyl isobutyrate	97-62-1	13: 43 71 116 41 88 73 89 42 39 101 117 72 70
6	Floral, fruity	17	6.91	0.08	4	Ethyl butyrate	105-54-4	17: 71 88 43 73 41 89 60 42 101 70 45 39 61 116 38 47 37
7	Solvent	18	7.2	0.07	4	Unknown		
8	Over ripe fruit 1	23	7.56	0.09	4	Unknown		
9	Over ripe fruit 2	18	8	0.16	4	Not detected		
10	Fruity 2	121	8.27	0.08	32	Ethyl isovalerate	108-64-5	6: 88 85 60 115 87 89
11	Banana	261	9.04	0.08	32	Isoamyl acetate	123-92-2	20: 43 70 55 87 61 41 42 73 69 39 71 88 58 56 44 57 85 45 53 54
12	Unknown pleasant	32	11.32	0.09	32	Unknown		
13	Fruity 3	38	12.22	0.1	4	Not detected		
14	Garlic	123	12.83	0.11	32	Not detected		
15	Unknown neutral 2	9	15.18	0.09	4	2-Nonanone	821-55-6	9: 58 43 59 71 57 82 127 84 100
16	Unknown unpleasant	132	15.7	0.1	32	Unknown		
17	Rose	76	15.91	0.16	32	Unknown		
18	Matchstick	71	16.7	0.08	32	Unknown		
19	Cut grass	327	17.06	0.2	32	Ethyl octanoate	106-32-1	20: 88 101 127 57 70 73 55 60 41 129 61 43 115 89 42 69 143 83 39 45
20	Barnyard	39	18.43	0.16	32	Unknown		
21	Mint	28	19.3	0.07	32	Unknown		
22	Unknown neutral 3	214	19.97	0.07	32	Phenethyl alcohol	60-12-8	10: 91 92 122 65 77 78 90 50 104 102
23	Strawberry 1	21	21.49	0.38	4	Ethyl decanoate	110-38-3	20: 88 101 155 157 70 73 55 41 43 61 69 60 89 115 57 71 143 83 200 42
24	Strawberry 2	393	22.01	0.16	32	Octanoic acid	124-07-2	20: 43 41 55 115 39 45 42 74 56 53 127 116 51 79 75 47 128 65 63 50
25	Tomato 1	146	24.32	0.02	32	Unknown		
26	Unknown neutral 4	194	24.78	0.02	32	Unknown		
27	Unknown neutral 5	132	25.27	0.02	32	Decanoic acid	334-48-5	20: 73 60 129 41 55 71 57 43 69 87 83 115 61 143 84 39 112 45 56 42
**Variety: Brianna; Harvest Time: 25 September 2015; Sample Number: 2**								
1	Rotten eggs	63	2.54	0.03	32	Not detected		
2	Alcoholic	264	3.2	0.19	32	Ethanol	64-17-5	
3	Butterscotch 1	3	4.2	0.38	1	Not detected		
4	Butterscotch 2	16	4.81	0.08	8	Not detected		
5	Body odor	4	5.31	0.08	4	Isobutyl alcohol	78-83-1	13: 43 33 41 42 39 74 55 56 57 53 75 49 54
6	Honey, caramel	144	5.85	0.01	32	Ethyl isobutyrate	97-62-1	11: 43 71 88 116 89 42 73 101 39 72 38
7	Floral, fruity	222	7.02	-0.03	32	Ethyl butyrate	105-54-4	19: 71 88 43 73 60 89 41 70 42 101 61 39 116 72 55 102 57 90 74
8	Solvent	114	7.3	-0.03	32	Unknown		
9	Over ripe fruit 2	122	8.04	0.06	32	Not detected		
10	Over ripe fruit 2	80	8.23	-0.07	32	Isoamyl alcohol	123-51-3	20: 88 101 155 157 70 73 55 41 43 61 69 60 89 115 57 71 143 83 200 42
11	Banana	91	9.19	-0.07	16	Isoamyl acetate	123-92-2	4: 56 43 55 57
12	Unknown pleasant	10	11.32	0.09	16	Not detected		
13	Fruity 3	45	12.36	-0.04	8	Unknown		
14	Garlic	61	12.83	0.11	32	Not detected		
15	Unknown unpleasant	37	15.7	0.1	8	Unknown		
16	Rose	1	15.91	0.16	1	Unknown		
17	Matchstick	33	16.7	0.08	32	Unknown		
18	Cut grass	96	17.14	0.12	16	Not detected		
19	Barnyard	3	18.43	0.16	8	Unknown		
20	Mint	39	19.3	0.07	32	Propyl octanoate	624-13-5	9: 69 121 190 105 120 77 122 79 145
21	Fruity 4	45	19.71	0.01	16	Unknown		
22	Unknown neutral 3	140	20.02	0.02	32	Phenethyl alcohol	60-12-8	20: 91 92 122 65 51 39 93 77 63 78 89 103 123 50 104 62 90 52 64 66
23	Strawberry 1	257	21.54	0.33	32	Ethyl decanoate	110-38-3	9: 106 105 77 51 52 76 75 37 49
24	Strawberry 2	12	22.05	0.12	1	Octanoic acid	124-07-2	16: 55 69 70 56 84 83 43 41 112 68 67 98 111 57 97 82
25	Tomato 1	179	24.32	0.02	32	Unknown		
26	Tomato 2	196	24.78	0.02	32	Unknown		
27	Unknown neutral 5	180	25.27	0.02	32	Decanoic acid	334-48-5	20: 73 60 129 57 43 55 41 71 69 87 83 61 84 39 143 74 42 45 112 56
**Variety: Frontenac gris; Harvest Time: 24 September 2015; Sample Number: 1**								
1	Rotten eggs, sulfury	7	2.54	2.04	2	Not detected		
2	Alcoholic	5	3.33	0.06	1	Ethanol	64-17-5	17: 45 46 43 42 47 41 44 33 40 48 77 49 61 39 104 34 96
3	Butterscotch	2	4.32	0.57	1	Dimethylamine	124-40-3	2: 44 40
4	Body odor	0	5.31	0.08	1	Isobutyl alcohol	78-83-1	4: 43 41 42 56
5	Honey, caramel, butterscotch	333	5.85	0.01	32	Ethyl isobutyrate	97-62-1	11: 43 71 41 116 88 73 89 42 39 55 53
6	Floral, fruity	11	6.99	0	2	Ethyl butyrate	105-54-4	20: 71 88 43 73 41 60 89 42 101 70 45 39 61 116 72 55 44 90 74 87
7	Solvent	5	7.27	0	1	Unknown		
8	Over ripe	3	8.01	0.09	1	Not detected		
9	Fruity 1	14	8.15	0.2	2	Not detected		
10	Banana	9	9.08	0.04	1	Isoamyl acetete	123-92-2	20: 43 70 55 87 61 41 42 73 69 39 71 88 58 56 57 85 45 53 54 115
11	Unknown pleasant 1	0	11.32	0.09	1	Unknown		
12	Fruity	2	12.29	0.03	1	Ethyl hexanoate	123-66-0	20: 88 99 43 101 60 70 73 71 41 61 55 42 115 45 39 87 69 117 89 100
13	Garlic	1	12.83	0.11	1	Not detected		
14	Unknown unpleasant 2	19	15.7	0.1	2	Methyl octanoate	111-11-5	11: 74 87 127 75 115 59 101 97 83 129 67
15	Cut grass, fruity	9	17.15	0.11	1	Ethyl octanoate	106-32-1	20: 88 101 127 57 73 70 60 55 41 61 43 129 115 89 42 69 143 45 83 39
16	Floral	5	19.5	0.54	1	Unknown		
17	Strawberry, honey	282	21.45	0.72	32	Ethyl decanoate	110-38-3	20: 88 101 155 157 73 70 55 41 43 60 61 69 89 115 57 71 143 83 42 84
18	Strawberry	11	21.98	0.2	2	Octanoic acid	124-07-2	20: 60 73 43 101 55 41 84 85 87 61 115 45 39 42 57 56 74 83 59 53
19	Tomato	1	24.78	0.02	1	Unknown		
20	Unknown neutral 2	0	25.27	0.02	1	Decanoic acid	334-48-5	20: 60 73 129 71 57 87 112 172 115 45 110 39 130 59 82 113 68 72 173 44
**Variety: Frontenac gris; Harvest Time: 24 September 2015; Sample Number: 2**								
1	Rotten eggs, sulfury	67	2.59	0.08	32	Not detected		
2	Alcoholic	347	3.39	0.13	32	Ethanol	64-17-5	16: 45 46 43 42 47 41 44 33 40 48 77 49 39 78 61 79
3	Honey, caramel, butterscotch	336	5.86	0.1	32	Ethyl isobutyrate	97-62-1	10: 43 71 88 73 89 39 72 101 57 56
4	Honey	64	6.53	0.04	32	Isobutyl acetate	110-19-0	9: 43 56 73 61 57 86 74 58 53
5	Unknown pleasant	259	7.09	0.07	32	Ethyl butyrate	105-54-4	20: 71 88 43 41 73 60 89 70 42 101 45 39 61 116 72 55 44 59 90 69
6	Solvent	213	7.38	0.07	32	Unknown		
7	Body odor	220	7.68	0.14	32	Isoamyl alcohol	123-51-3	15: 55 70 42 43 39 45 71 46 44 53 40 54 35 60 52
8	Fruity 1	269	8.2	0.09	32	Ethyl methylbutyrate	7452-79-1	6: 102 85 74 87 115 103
9	Fruity 2	85	8.4	0.09	32	Ethyl isovalerate	108-64-5	10: 88 85 60 87 61 115 86 59 89 130
10	Banana	306	9.16	0.08	32	Isoamyl acetete	123-92-2	20: 43 70 55 87 61 41 42 73 69 39 71 88 44 58 56 57 85 45 53 54
11	Woody 1	27	10.31	0.06	32	Ethyl lactate	97-64-3	6: 45 75 43 47 61 74
12	Vinegar	37	11.45	0.06	32	Acetic acid	64-19-7	6: 43 45 60 42 41 44
13	Cereal	100	11.75	0.39	32	Unknown		
14	Fruity	306	12.43	0.11	32	Ethyl hexanoate	123-66-0	20: 88 99 43 101 60 70 73 71 61 41 55 42 115 45 39 87 69 117 89 102
15	Garlic	102	12.89	0.11	32	Not detected		
16	Mushroom	52	14.95	0.06	32	Unknown		
17	Sweaty	250	15.74	0.45	32	Methyl octanoate	111-11-5	6: 74 87 115 59 98 84
18	Match, sulfury	88	16.74	0.08	32	Not detected		
19	Cut grass, fruity	340	17.26	0.19	32	Ethyl octanoate	106-32-1	20: 88 101 127 57 73 70 60 55 41 61 43 129 115 89 42 69 143 45 83 39
20	Woody 2	63	18.23	0.12	32	Unknown		
21	Rose 2	249	20.05	0.3	32	Phenethyl alcohol	60-12-8	20: 91 92 122 65 39 51 78 89 103 104 123 50 62 52 64 66 38 41 76 121
22	Strawberry, honey	266	21.45	0.3	32	Ethyl decanoate	110-38-3	20: 88 101 155 157 73 70 55 41 43 60 61 69 89 57 115 71 143 83 42 84
23	Strawberry	422	22.1	0.11	32	Octanoic acid	124-07-2	20: 60 73 43 41 55 101 85 84 87 115 61 45 69 39 42 57 56 74 83 82
24	Carrots, woody	113	22.61	0.69	32	Unknown		
25	Fecal	55	23.4	0.02	32	Unknown		
**Variety: Frontenac gris; Harvest Time: 1 October 2015; Sample Number: 1**								
1	Rotten eggs, sulfury	58	2.59	0.08	32	Not detected		
2	Alcoholic	361	3.39	0.13	32	Ethanol	64-17-5	16: 45 46 43 42 47 41 44 40 33 77 39 49 61 56 115 129
3	Honey, caramel, butterscotch	431	5.86	0.1	32	Ethyl isobutyrate	97-62-1	11: 43 71 41 88 116 89 72 44 87 55 70
4	Honey	122	6.53	0.04	32	Isobutyl acetate	110-19-0	9: 43 56 73 41 39 71 61 57 37
5	Unknown pleasant	307	7.09	0.07	32	Ethyl butyrate	105-54-4	19: 71 88 43 73 41 60 70 101 42 45 61 39 116 55 44 57 87 69 117
6	Solvent	291	7.38	0.07	32	Unknown		
7	Body odor	277	7.68	0.14	32	Isoamyl alcohol	123-51-3	16: 55 70 42 41 43 39 44 53 40 54 38 50 47 37 72 36
8	Fruity 1	314	8.2	0.09	32	Ethyl methylbutyrate	7452-79-1	7: 102 85 87 115 103 73 75
9	Fruity 2	112	8.4	0.09	32	Ethyl isovalerate	108-64-5	7: 88 85 60 87 115 59 103
10	Banana	337	9.16	0.08	32	Isoamyl acetete	123-92-2	20: 43 70 55 87 61 41 42 73 69 39 71 58 56 88 44 57 85 45 53 54
11	Woody 1	55	10.31	0.06	32	Ethyl lactate	97-64-3	5: 45 75 44 47 56
12	Vinegar	57	11.45	0.06	32	Acetic acid	64-19-7	4: 43 45 60 42
13	Cereal	138	11.75	0.39	32	Unknown		
14	Fruity	333	12.43	0.11	32	Ethyl hexanoate	123-66-0	20: 88 99 43 101 60 70 73 71 41 61 55 42 115 45 39 87 69 117 89 74
15	Garlic	129	12.89	0.11	32	Not detected		
16	Mushroom	55	14.95	0.06	32	Unknown		
17	Sweaty	250	15.74	0.45	32	Methyl octanoate	111-11-5	15: 74 87 127 43 57 55 115 59 41 75 101 129 84 39 98
18	Rose 1	27	16.44	0.05	32	Unknown		
19	Match, sulfury	136	16.74	0.08	32	Not detected		
20	Cut grass, fruity	348	17.26	0.19	32	Ethyl octanoate	106-32-1	20: 88 101 127 57 73 70 55 60 41 61 129 43 115 89 42 69 143 83 45 39
21	Woody 2	52	18.23	0.12	32	Unknown		
22	Rose 2	290	20.05	0.3	32	Phenethyl alcohol	60-12-8	20: 91 92 122 65 51 77 39 93 63 78 89 103 50 62 90 52 66 79 64 102
23	Strawberry, honey	268	21.45	0.3	32	Ethyl decanoate	110-38-3	20: 88 101 155 157 73 70 55 41 43 60 61 69 115 89 57 71 143 83 42 84
24	Strawberry	458	22.1	0.11	32	Octanoic acid	124-07-2	20: 60 73 43 55 101 41 85 84 87 69 115 61 45 39 42 57 56 74 83 82
25	Carrots, woody	156	22.61	0.69	32	Unknown		
26	Fecal	61	23.4	0.02	32	Unknown		
**Variety: Frontenac gris; Harvest Time: 01 October 2015; Sample Number: 2**								
1	Rotten eggs, sulfury	75	2.59	0.08	32	Not detected		
2	Alcoholic	391	3.39	0.13	32	Ethanol	64-17-5	14: 45 46 43 42 47 41 44 40 33 77 49 39 38 78
3	Honey, caramel, butterscotch	423	5.86	0.1	32	Ethyl isobutyrate	97-62-1	13: 43 71 41 88 116 73 42 89 101 55 72 90 57
4	Honey	87	6.53	0.04	32	Isobutyl acetate	110-19-0	12: 43 56 73 41 71 39 61 57 55 86 44 38
5	Unknown pleasant	324	7.09	0.07	32	Ethyl butyrate	105-54-4	20: 71 88 43 41 73 60 89 42 70 101 45 39 61 72 55 44 57 40 87 69
6	Solvent	337	7.38	0.07	32	Unknown		
7	Body odor	359	7.68	0.14	32	Isoamyl alcohol	123-51-3	20: 55 70 42 41 43 39 45 71 46 53 40 54 51 50 72 49 35 65 86 48
8	Fruity 1	345	8.2	0.09	32	Ethyl methylbutyrate	7452-79-1	6: 102 85 87 74 103 115
9	Fruity 2	122	8.4	0.09	32	Ethyl isovalerate	108-64-5	7: 88 85 60 115 87 73 86
10	Banana	382	9.16	0.08	32	Isoamyl acetate	123-92-2	20: 43 70 55 87 61 41 42 73 69 39 71 88 56 58 44 57 85 53 45 54
11	Woody 1	49	10.31	0.06	32	Ethyl lactate	97-64-3	6: 45 75 47 46 103 89
12	Vinegar	38	11.45	0.06	32	Acetic acid	64-19-7	5: 45 43 60 42 47
13	Cereal	152	11.75	0.39	32	Unknown		
14	Fruity	419	12.43	0.11	32	Ethyl hexanoate	123-66-0	20: 88 99 43 101 60 70 73 71 61 41 55 42 115 45 39 87 69 117 89 74
15	Garlic	207	12.89	0.11	32	Not detected		
16	Mushroom	48	14.95	0.06	32	Unknown		
17	Sweaty	372	15.74	0.45	32	Methyl octanoate	111-11-5	14: 74 87 127 55 57 101 115 59 75 84 69 98 85 128
18	Rose 1	34	16.44	0.05	32	Unknown		
19	Match, sulfury	153	16.74	0.08	32	Not detected		
20	Cut grass, fruity	417	17.26	0.19	32	Ethyl octanoate	106-32-1	20: 88 101 127 57 73 70 55 60 41 61 129 43 115 89 42 69 143 83 45 39
21	Woody 2	71	18.23	0.12	32	Unknown		
22	Rose 2	321	20.05	0.3	32	Phenethyl alcohol	60-12-8	20: 91 92 122 65 39 51 77 93 89 78 103 104 50 123 62 90 52 64 66 38
23	Strawberry, honey	321	21.45	0.3	32	Ethyl decanoate	110-38-3	20: 88 101 155 157 73 70 55 41 43 60 61 69 89 115 57 71 143 83 42 84
24	Strawberry	517	22.1	0.11	32	Octanoic acid	124-07-2	20: 60 73 43 101 55 41 85 84 87 69 115 61 45 39 42 57 56 74 83 59
25	Carrots, woody	178	22.61	0.69	32	Unknown		
26	Fecal	58	23.4	0.02	32	Unknown		
**Variety: Frontenac gris; Harvest Time: 09 October 2015; Sample Number: 1**								
1	Rotten eggs, sulfury	78	2.59	0.08	32	Not detected		
2	Alcoholic	355	3.39	0.13	32	Ethanol	64-17-5	17: 45 46 43 42 47 41 44 40 33 77 49 39 61 78 55 34 53
3	Unknown neutral 1	5	4.16	0.03	2	Not detected		
4	Honey, caramel, butterscotch	378	5.86	0.1	32	Ethyl isobutyrate	97-62-1	13: 43 71 41 116 88 73 89 101 39 42 72 70 117
5	Honey	60	6.53	0.04	32	Isobutyl acetate	110-19-0	7: 43 56 73 39 57 61 86
6	Unknown pleasant	296	7.09	0.07	32	Ethyl butyrate	105-54-4	20: 71 88 43 73 41 60 89 70 42 101 45 39 61 72 116 55 44 40 57 38
7	Solvent	296	7.38	0.07	32	Unknown		
8	Body odor	318	7.68	0.14	32	Isoamyl alcohol	123-51-3	20: 55 70 42 41 43 39 45 69 71 46 44 40 38 51 50 37 73 49 86 85
9	Fruity 1	289	8.2	0.09	32	Ethyl methylbutyrate	7452-79-1	6: 102 85 74 115 87 103
10	Fruity 2	170	8.4	0.09	32	Ethyl isovalerate	108-64-5	8: 88 85 60 61 87 59 73 103
11	Banana	374	9.16	0.08	32	Isoamyl acetate	123-92-2	20: 43 70 55 87 61 41 42 73 69 39 71 56 88 44 58 57 85 45 53 54
12	Woody 1	47	10.31	0.06	32	Ethyl lactate	97-64-3	2: 45 75
13	Vinegar	35	11.45	0.06	32	Acetic acid	64-19-7	7: 45 43 60 42 44 47 72
14	Cereal	145	11.75	0.39	32	Unknown		
15	Fruity	416	12.43	0.11	32	Ethyl hexanoate	123-66-0	20: 88 99 43 101 60 70 73 71 61 41 55 42 115 45 39 87 69 117 89 102
16	Garlic	168	12.89	0.11	32	Not detected		
17	Mushroom	61	14.95	0.06	32	Unknown		
18	Sweaty	384	15.74	0.45	32	Methyl octanoate	111-11-5	9: 74 87 115 57 59 75 84 98 83
19	Rose 1	39	16.44	0.05	32	Unknown		
20	Match, sulfury	134	16.74	0.08	32	Not detected		
21	Cut grass, fruity	381	17.26	0.19	32	Ethyl octanoate	106-32-1	20: 88 101 127 57 73 70 55 60 41 61 129 43 115 42 89 69 143 83 45 39
22	Woody 2	78	18.23	0.12	32	Unknown		
23	Rose 2	319	20.05	0.3	32	Phenethyl alcohol	60-12-8	20: 91 92 122 65 77 51 39 63 93 78 89 103 123 104 50 90 62 64 79 66
24	Strawberry, honey	336	21.45	0.3	32	Ethyl decanoate	110-38-3	20: 88 101 155 157 73 70 55 41 43 60 61 69 115 89 57 71 143 83 42 85
25	Strawberry	457	22.1	0.11	32	Octanoic acid	124-07-2	20: 60 73 43 101 55 41 85 84 87 115 69 61 39 45 42 57 56 74 83 97
26	Carrots, woody	155	22.61	0.69	32	Unknown		
27	Fecal	97	23.4	0.02	32	Unknown		
**Variety: Frontenac gris; Harvest Time: 9 October 2015; Sample Number: 2**								
1	Rotten eggs, sulfury	61	2.59	0.08	32	Not detected		
2	Alcoholic	304	3.39	0.13	32	Ethanol	64-17-5	15: 45 46 43 42 47 41 44 33 40 48 77 49 39 61 34
3	Unknown neutral 1	0	4.15	0.02	1	Not detected		
4	Honey, caramel, butterscotch	320	5.86	0.1	32	Ethyl isobutyrate	97-62-1	8: 43 71 41 88 116 73 89 72
5	Honey	41	6.53	0.04	32	Isobutyl acetate	110-19-0	14: 43 56 73 41 39 71 57 86 74 55 44 60 58 101
6	Unknown pleasant	268	7.09	0.07	32	Ethyl butyrate	105-54-4	19: 71 88 43 73 41 60 89 42 70 45 39 61 55 44 40 69 38 74 102
7	Solvent	292	7.38	0.07	32	Unknown		
8	Body odor	349	7.68	0.14	32	Isoamyl alcohol	123-51-3	16: 55 70 42 43 39 69 71 46 53 54 50 59 60 72 52 65
9	Fruity 1	23	8.2	0.09	2	Ethyl methylbutyrate	7452-79-1	6: 102 85 74 115 87 103
10	Fruity 2	244	8.4	0.09	32	Ethyl isovalerate	108-64-5	8: 88 85 60 61 87 115 59 73
11	Banana	373	9.16	0.08	32	Isoamyl acetate	123-92-2	20: 43 70 55 87 61 41 42 73 69 39 71 88 56 44 58 57 85 45 53 54
12	Woody 1	32	10.31	0.06	32	Ethyl lactate	97-64-3	3: 45 75 76
13	Vinegar	30	11.45	0.06	32	Acetic acid	64-19-7	7: 43 45 60 42 41 61 47
14	Cereal	147	11.75	0.39	32	Unknown		
15	Fruity	389	12.43	0.11	32	Ethyl hexanoate	123-66-0	20: 88 99 43 101 60 70 73 71 61 41 55 42 115 45 39 87 69 117 89 100
16	Garlic	145	12.89	0.11	32	Not detected		
17	Mushroom	63	14.95	0.06	32	Unknown		
18	Sweaty	358	15.74	0.45	32	Methyl octanoate	111-11-5	11: 74 87 127 75 115 59 101 97 83 129 67
19	Rose 1	132	16.44	0.05	32	Unknown		
20	Match, sulfury	183	16.74	0.08	32	Not detected		
21	Cut grass, fruity	394	17.26	0.19	32	Ethyl octanoate	106-32-1	20: 88 101 127 57 73 70 55 60 41 61 129 43 115 89 42 69 143 83 45 39
22	Woody 2	102	18.23	0.12	32	Unknown		
23	Rose 2	321	20.05	0.3	32	Phenethyl alcohol	60-12-8	20: 91 92 122 65 39 51 77 63 93 78 89 103 104 50 90 52 66 41 38 61
24	Strawberry, honey	395	21.45	0.3	32	Ethyl decanoate	110-38-3	20: 88 101 155 157 73 70 55 41 43 60 61 69 89 115 57 71 143 83 42 85
25	Strawberry	524	22.1	0.11	32	Octanoic acid	124-07-2	20: 60 73 43 55 101 41 85 84 87 69 61 115 39 45 42 57 56 74 83 59
26	Carrots, woody	155	22.61	0.69	32	Unknown		
27	Fecal	117	23.4	0.02	32	Unknown		

* OD = Odor dilution (defined in Materials and Methods).

## References

[1] Wine America Economic Impact Reports. http://wineamerica.org/impact.

[2] White M.L. History of the Institute. Proceedings of the Midwest Grape and Wine Industry Institute Advisory Board Meeting Minutes, Interpower Building.

[3] Tuck B., Gartner W., Appiah G. (2015). Economic Contribution of Vineyards and Wineries of the North. https://conservancy.umn.edu/bitstream/handle/11299/197808/2015-economic-contribution-wineries-and-grapes.pdf?sequence=1&isAllowed=y.

[4] Parker M., Capone D.L., Francis I.L., Herderich M.J. (2018). Aroma precursors in grapes and wine: Flavor release during wine production and consumption. J. Agric. Food Chem..

[5] Gonzalez R., Morales P. (2017). Wine secondary aroma: Understanding yeast production of higher alcohols. Microb. Biotechnol..

[6] Rapp A. (1998). Volatile flavour of wine: Correlation between instrumental analysis and sensory perception. Nahrung.

[7] Acree T., Arn H. Flavornet and Human Odor Space. http://www.flavornet.org.

[8] LRI & Odour Database. http://www.odour.org.uk/index.html.

[9] Boulton R.B., Singleton V.L., Bisson L.F., Kunkee R.E. (1996). Principles and Practices of Winemaking.

[10] Jordão A.M., Vilela A., Cosme F. (2015). From sugar of grape to alcohol of wine: Sensorial impact of alcohol in wine. Beverages.

[11] Okie W.R. (2004). Register of new fruit and nut varieties, List 42. HortScience.

[12] Luby J., Hemstad P. (2006). Grape Plant Named ‘Frontenac Gris’. Regents of the University of Minnesota, Minneapolis, MN (US), assignee. U.S. Patent.

[13] Frontenac Gris Wine University of Minnesota Cold Hardy Grapes. http://www.grapes.umn.edu/gris/enology.html.

[14] Atucha A., Hedtcke J., Workmaster B.A. (2018). Evaluation of cold-climate interspecific hybrid wine grape cultivars for the upper Midwest. J. Am. Pomol. Soc..

[15] Cai L., Rice S., Koziel J.A., Dharmadhikari M. (2017). Development of an automated method for aroma analysis of red wines from cold-hardy grapes using simultaneous solid-phase microextraction—Multidimensional gas chromatography—Mass spectrometry—Olfactometry. Separations.

[16] Pawliszyn J. (2009). Handbook of Solid Phase Microextraction.

[17] Wang X., Tao Y., Wu Y., An R., Yue Z. (2017). Aroma compounds and characteristics of noble-rot wines of Chardonnay grapes artificially botrytized in the vineyard. Food Chem..

[18] Bordiga M., Rinaldi M., Locatelli M., Piana G., Travaglia F., Coïsson J.D., Arlorio M. (2013). Characterization of Muscat wines aroma evolution using comprehensive gas chromatography followed by a post-analytic approach to 2D contour plots comparison. Food Chem..

[19] Wenlai F., Yan X., Wenguang Jiang A.J.L., Jiming L. (2010). Identification and quantification of impact aroma compounds in 4 nonfloral *Vitis vinifera* varieties grapes. J. Food Sci..

[20] Sun Q., Gates M.J., Lavin E.H., Acree T.E., Sacks G.L. (2011). Comparison of odor-active compounds in grapes and wines from vitis vinifera and non-foxy American grape species. J. Agric. Food Chem..

[21] The Northern Grapes Project: Viticulture, Enology and Marketing for Cold-Hardy Grapes. http://northerngrapesproject.org.

[22] Rice S., Lutt N., Koziel J.A., Dharmadhikari M., Fennell A. (2018). Determination of selected aromas in Marquette and Frontenac wine using headspace-SPME coupled with GC-MS and simultaneous olfactometry. Separations.

[23] Cai L., Rice S., Koziel J.A., Jenks W.S., van Leeuwen J.H. (2016). Further purification of food-grade alcohol to make a congener-free product. J. Inst. Brew..

[24] The Good Scents Company Information System. http://www.thegoodscentscompany.com/.

[25] Onuki S., Koziel J.A., Jenks W.S., Cai L., Rice S., van Leeuwen J.H. (2016). Optimization of extraction parameters for quantification of fermentation volatile by-products in industrial ethanol with solid-phase microextraction and gas chromatography. J. Inst. Brew..

[26] Rice S., Koziel J.A. (2015). Characterizing the smell of marijuana by odor impact of volatile compounds: An application of simultaneous chemical and sensory analysis. PLoS ONE.

[27] Rice S., Koziel J.A. (2015). Odor impact of volatiles emitted from marijuana, cocaine, heroin and their surrogate scents. Data Brief.

[28] Rice S., Koziel J.A. (2015). The relationship between chemical concentration and odor activity value explains the inconsistency in making a comprehensive surrogate scent training tool representative of illicit drugs. Forensic Sci. Int..

[29] Reboredo-Rodríguez P., González-Barreiro C., Rial-Otero R., Cancho-Grande B., Simal-Gándara J. (2015). Effects of sugar concentration processes in grapes and wine aging on aroma compounds of sweet wines—A review. Crit. Rev. Food Sci. Nutr..

[30] Camper C. Chateau Stripmine—Brianna Parentage. http://chateaustripmine.info/Parentage/Brianna.gif.

[31] Camper C. Chateau Stripmine—Frontenac Parentage. http://chateaustripmine.info/Parentage/Frontenac.gif.

[32] Jackson R.S. (2008). Wine Science Principles and Applications.

[33] González-Álvarez M., Noguerol-Pato R., González-Barreiro C., Cancho-Grande B., Simal-Gándara J. (2013). Sensory quality control of young vs. aged sweet wines obtained by the techniques of both postharvest natural grape dehydration and fortification with spirits during vinification. Food Anal. Method.

[34] Noguerol-Pato R., González-Álvarez M., González-Barreiro C., Cancho-Grande B., Simal-Gándara J. (2013). Evolution of the aromatic profile in Garnacha Tintorera grapes during raisining and comparison with that of the naturally sweet wine obtained. Food Chem..

[35] Mauricio J.C., Moreno J., Zea L., Ortega J.M., Medina M. (1997). The effects of grape must fermentation conditions on volatile alcohols and esters formed by *Saccharomyces cerevisiae*. J. Sci. Food Agric..

[36] Simpson R.F., Miller G.C. (1984). Aroma composition of Chardonnay wine. Vitis.

[37] Straus C.R., Wilson B., Anderson R., Williams P.J. (1987). Application of droplet countercurrent chromatography to the analysis of conjugated forms of terpenoids, phenols, and other constituents of grape juice. J. Agric. Food Chem..

[38] Ferreira V., Pena C., Escudero C.L., Fernandez P., Cacho J., Benedito C., Collar C., Martinez M., Morel J. (1993). Method for the HPLC prefractionation of wine flavour extracts. Part II—Sensory aspects. Profiling wine aroma. Progress in Food Fermentation.

[39] Marais J., Pool H.J. (1980). Effect of storage time and temperature on the volatile composition and quality of dry white table wines. Vitis.

[40] Marais J. (1983). Terpenes in the Aroma of Grapes and Wines; A review. S. Afr. J. Enol. Vitic..

[41] Cullere L., Escudero E.C., Campo E., Cacho J., Ferreira V. (2009). Multidimensional gas chromatography-mass spectrometry determination of 3-alkyl-2-methoxypyrazines in wine and must. A comparison of solid-phase extraction and headspace solid-phase extraction methods. J. Chromatogr. A.

[42] Kolor M.K. (1983). Identification of an important new flavor compound in Concord grape, ethyl 3-mercaptopropionate. J. Agric. Food Chem..

[43] Tominaga T., Niclass Y., Frerot E., Dubourdiue D. (2006). Stereoisomeric distribution of 3-mercaptohexan-1-ol and 3-mercaptohexyl acetate in dry and sweet white wines made from *Vitis vinifera* (Var. Sauvignon Blanc and Semillon). J. Agric. Food Chem..

[44] Guth H. (1997). Identification of character impact odorants of different white wine varieties. J. Agric. Food Chem..

[45] Winton W., Ough C.S., Singleton V.L. (1975). Relative distinctiveness of varietal wines estimated by the ability of trained panelists to name the grape variety correctly. Am. J. Enol. Vitic..

[46] González-Barreiro C., Rial-Otero R., Cancho-Grande B., Simal-Gándara J. (2015). Wine aroma compounds in grapes: A critical review. Crit. Rev. Food Sci. Nutr..

[47] Mansfield A.K., Vickers Z.M. (2009). Characterization of the aroma of red Frontenac table wines by descriptive analysis. Am. J. Enol. Vitic..

[48] Mansfield A.K., Schirle-Keller J.P., Reineccius G.A. (2011). Identification of odor-impact compounds in red table wines produced from Frontenac grapes. Am. J. Enol. Vitic..

[49] Pedneault K., Dorais M., Angers P. (2013). Flavor of cold-hardy grapes: Impact of berry maturity and environmental conditions. J. Agric. Food Chem..

[50] Slegers A., Angers P., Ouillet E., Truchon T., Pedneault K. (2015). Volatile compounds from grape skin, juice and wine from five interspecific hybrid grape cultivars grown in Quebec (Canada) for wine production. Molecules.

[51] Brady J.M. Descriptive Analysis of *Frontenac gris* and Brianna Wine Grape and Wine Varieties. Retrieved from the University of Minnesota Digital Conservancy. http://hdl.handle.net/11299/194662.

[52] Sanchez-Palomo E., Diaz-Maroto M.C., Gonzalez-Vinas M.A., Soriano-Perez A., Perez-Coello M.S. (2005). Aroma profile of wines from Albillo and Muscat grape varieties at different stages of ripening. Food Control.

[53] Bindon K., Varela C., Kennedy J., Holt H., Herderich M. (2013). Relationships between harvest time and wine composition in *Vitis vinifera* L. cv. Cabernet Sauvignon 1. Grape and wine chemistry. Food Chem..

[54] Gomez-Miguez M.J., Gomez-Miguez M., Vicario I.M., Heredia F.J. (2007). Assessment of colour and aroma in white wines vinifications: Effects of grape maturity and soil type. J. Food Eng..

[55] Vilanova M., Genisheva Z., Bescansa L., Masa A., Oliveira J. (2012). Changes in free and bound fractions of aroma compounds of four *Vitis vinifera* cultivars at the last ripening stages. Phytochemistry.

[56] Yuan F., Qian M. (2006). Quantification of selected aroma-active compounds in Pinot noir wines from different grape maturities. J. Agric. Food Chem..

[57] Yuan F., Qian M.C. (2016). Aroma potential in early- and late-maturity Pinot noir grapes evaluated by aroma extract dilution analysis. J. Agric. Food Chem..

[58] Jiang B., Zhang Z.-W. (2018). A Preliminary study of aroma composition and impact odorants of Cabernet Franc wines under different terrain conditions of the Loess Plateau Region (China). Molecules.

[59] Zhao P., Gao J., Qian M., Li H. (2017). Characterization of the key aroma compounds in Chinese Syrah wine by gas chromatography-olfactometry-mass spectrometry and aroma reconstitution studies. Molecules.

[60] Liu P.-H., Vrigneau C., Salmon T., Hoang D.A., Boulet J.-C., Jégou S., Marchal R. (2018). Influence of grape berry maturity on juice and base wine composition and foaming properties of sparkling wines from the Champagne region. Molecules.

[61] Ristic R., Boss P.K., Wilkinson K.L. (2015). Influence of fruit maturity at harvest on the intensity of smoke taint in wine. Molecules.

[62] Zhang P., Luo F., Howell K. (2017). Fortification and elevated alcohol concentration affect the concentration of rotundone and volatiles in *Vitis vinifera* cv. Shiraz Wine. Fermentation.

[63] Gonzalez-Alvarez M., Gonzalez-Barreiro C., Cancho-Grande B., Simall-Gandara J. (2011). Relationship between Godello white wine sensory properties and its aromatic fingerprinting obtained by GC-MS. Food Chem..

[64] Noguerol-Pato R., Gonzalez-Alvarez M., Gonzalez-Barreiro C., Cancho-Grande B., Simal-Gandara J. (2012). Aroma profile of Garnacha Tintorera-based sweet wines by chromatographic and sensorial analysis. Food Chem..

[65] Noguerol-Pato R., Gonzalez-Barreiro C., Cancho-Grande B., Santiago J.L., Martinez M.C., Simal-Gandara J. (2011). Aroma potential of Brancellao grapes from different cluster positions. Food Chem..

[66] Noguerol-Pato R., González-Barreiro C., Simal-Gándara J., Martínez M.C., Santiago J.L., Cancho-Grande B. (2012). Active odorants in Mouratón grapes from shoulders and tips into the bunch. Food Chem..

[67] Noguerol-Pato R., González-Barreiro C., Cancho-Grande B., Martínez M.C., Santiago J.L., Simal-Gándara J. (2012). Floral, spicy and herbaceous active odorants in Gran Negro berries from shoulders and tips into the cluster, and comparison with Brancellao and Mouratón varieties. Food Chem..

[68] Rice S., Koziel J.A., Dharmadhikari M., Fennell A. (2017). Evaluation of tannins and anthocyanins in Marquette, Frontenac, and St. Croix cold-hardy grape cultivars. Fermentation.

